# Linking Viscosity and Droplet Microstructure in Liquid Metal Composites via 3D MicroCT Analysis

**DOI:** 10.1002/smll.202512413

**Published:** 2026-01-12

**Authors:** Hugh P. Grennan, Ohnyoung Hur, Michael D. Bartlett

**Affiliations:** ^1^ Mechanical Engineering, Soft Materials and Structures Lab Virginia Tech Blacksburg VA 24061 USA; ^2^ Macromolecules Innovation Institute Virginia Tech Blacksburg VA 24061 USA

**Keywords:** liquid metal composites, micro‐computed tomography (microCT), processing‐structure relationships, rheology, soft electronics

## Abstract

Liquid metal (LM) composites offer unique combinations of compliance, conductivity, and functionality that enable applications in soft robotics, wearable devices, and flexible electronics. Realizing these capabilities requires a fundamental understanding of how processing influences microstructure, since droplet size, dispersion, and settling govern material properties. Here, rheological measurements are combined with micro‐computed tomography (microCT) imaging to uncover how uncured composite viscosity directs the formation of LM microstructures in elastomeric matrices. By systematically varying LM volume fraction (*ϕ* = 10%, 20%, 30%) and fumed silica (FS) weight fraction (*ψ* = 0%, 4%, 8%) while holding planetary mixing conditions constant, the role of rheology is isolated in shaping LM droplet populations. MicroCT analysis provides quantitative 3D characterization of thousands of droplets, enabling statistical evaluation of size distributions, spatial dispersion, and settling behavior. These findings are further analyzed by modeling LM droplet settling during polymer curing, enabling the prediction of microstructural homogeneity and providing a design tool for tailoring composite properties. This approach reveals how composite rheology dictates LM microstructure, which can be modified to achieve a ≈ 10^5^x increase in electrical conductivity upon indentation. These insights provide design guidelines for processing LM composites with tailored microstructures, advancing their performance in functional devices.

## Graphical abstract

This work connects rheology and microstructure in liquid metal composites through 3D microCT imaging. Quantitative analysis of thousands of droplets reveals how liquid metal and fumed silica contents dictate droplet size, settling, and dispersion, providing design principles for processing‐structure control and advancing high‐performance, multifunctional soft composites.

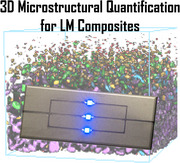

## 1 Introduction

Soft composites with embedded liquid metal (LM) microstructures exhibit unique combinations of stretchability,^[^ ^1^, ^2^ ^]^ electrical conductivity,^[^ ^3^, ^4^ ^]^ and thermal conductivity ^[^ ^5^, ^6^, ^7^ ^]^ that enable emerging technologies in soft robotics,^[^ ^8^, ^9^, ^10^, ^11^, ^12^ ^]^ wearable medical devices,^[^ ^13^, ^14^, ^15^ ^]^ and thermal interface materials.^[^ ^16^, ^17^ ^]^ The process by which LM is incorporated into the composite is crucial, since processing conditions directly influence the material microstructure. This, in turn, greatly impacts the properties of the LM composite, such as thermal and electrical conductivity^[^ ^7^, ^18^, ^19^, ^20^ ^]^ and dielectric integrity.^[^ ^21^, ^22^, ^23^ ^]^

For liquid–liquid emulsions, increasing the emulsification rate (via mixing) typically reduces droplet size due to enhanced breakup forces.^[^ ^24^, ^25^, ^26^ ^]^ Dispersion and integration of LM into uncured elastomer can involve sonication for sub‐micron droplets,^[^ ^27^, ^28^, ^29^, ^30^ ^]^ and overhead mixing or planetary mixing for microscale droplets.^[^ ^31^, ^32^, ^33^ ^]^ Because different processing approaches impose distinct shear environments, they strongly affect LM droplet size and dispersion. Consequently, tuning the rheology of uncured composites provides a route to mitigate or promote effects such as droplet sedimentation and droplet settling.^[^ ^34^, ^35^, ^36^, ^37^, ^38^ ^]^ For example, fumed silica (FS) increases the viscosity and shear‐thinning behavior of polydimethylsiloxane (PDMS)^[^ ^39^, ^40^ ^]^ which can resist LM droplet settling. Rheological modification is also critical in 3D printing and additive manufacturing approaches to control droplet elongation and printability. ^[^ ^41^, ^42^ ^]^ Therefore, the connection between rheology and microstructure in LM composites is critical to achieving tunable composite properties.

Characterizing droplet size and dispersion is essential for linking rheology to microstructure. Conventional methods such as optical microscopy and scanning electron microscopy are widely used,^[^ ^43^, ^44^ ^]^ and small‐angle X‐ray scattering (SAXS) can characterize nanoscale LM droplets.^[^ ^28^ ^]^ However, these approaches have limitations: SAXS and other scattering techniques are not applicable for micron‐scale droplets (> 20 µm in diameter),^[^ ^28^ ^]^ and microscopy is restricted to surface or cross‐sectional views. Heterogeneities such as droplet settling are therefore difficult to quantify in three dimensions using these techniques.^[^ ^45^ ^]^ Micro‐computed tomography (microCT) scanning offers a compelling alternative for visualizing and analyzing the LM 3D microstructure. While tomography techniques have been applied to study material microstructures, such as porosity and defects in additively manufactured parts^[^ ^46^, ^47^, ^48^ ^]^ and structural changes within Li‐ion batteries,^[^ ^49^ ^]^ the use of microCT for LM composites has largely been focused on visualization,^[^ ^50^, ^51^, ^52^, ^53^ ^]^ leaving significant opportunities to establish stronger links between processing, rheology, and microstructure.

**FIGURE 1:**
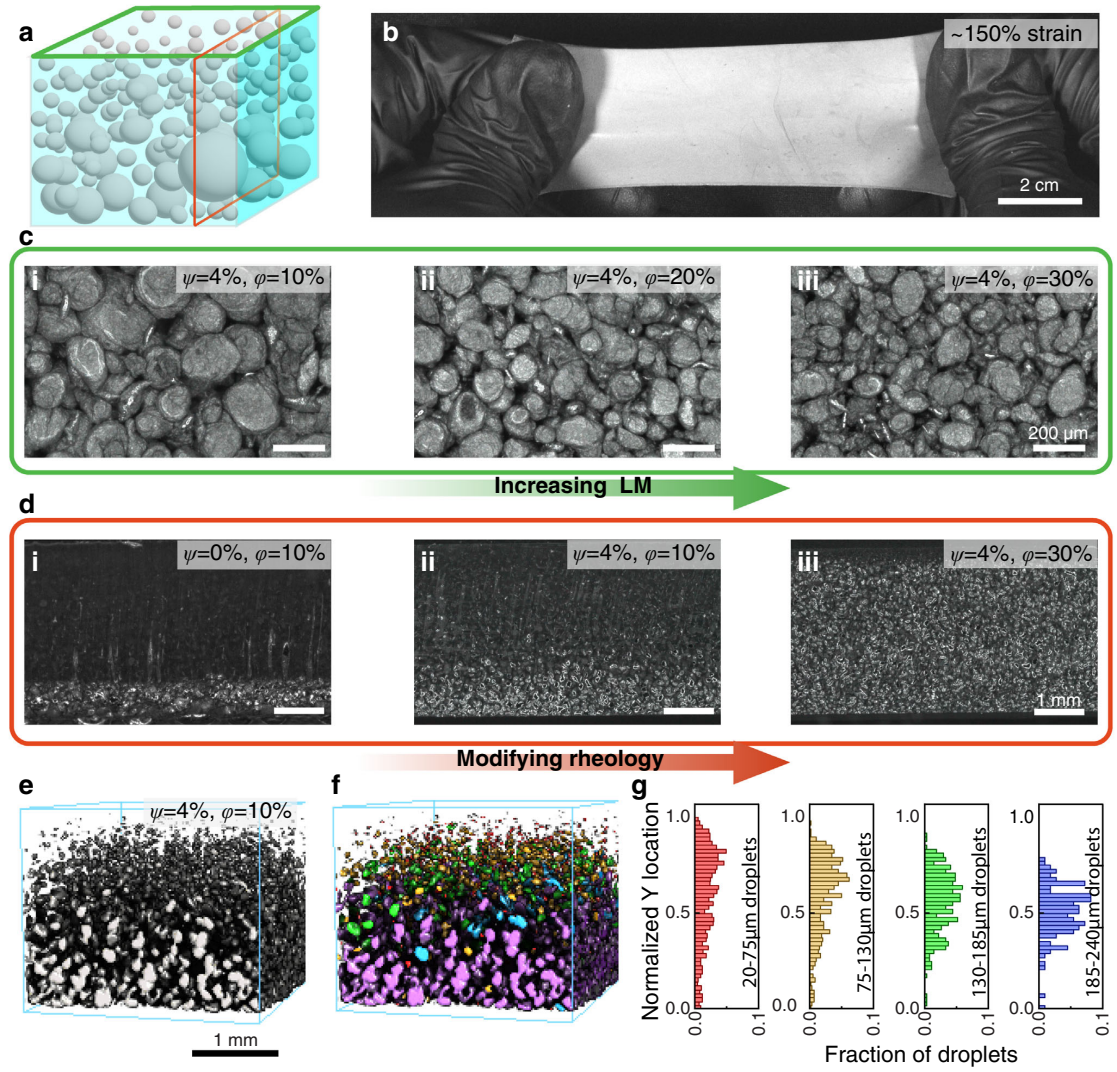
Visualizing the effects of modifying rheological properties on LM composite microstructure. a) Schematic representation of LM composite microstructure. b) Image of a bulk LM composite, strained to ≈ 150%. c) Optical micrographs of composite surfaces, demonstrating that LM volume fraction results in decreased LM droplet size. d) Optical micrographs of cross‐sectional images of LM composite samples, demonstrating that rheological modification results in increased LM droplet homogeneity. e) 3D rendering of raw microCT data. f) 3D rendering of segmented and analyzed microCT data, where droplets are colored by relative droplet size. g) Histograms denoting the Y‐location of subsets of droplets within an LM composite.

In this work, we combine rheological measurements with microCT imaging to uncover how uncured composite viscosity governs the size, dispersion, and settling of LM droplets in soft elastomeric composites (**Figure** **1**a,b). By systematically varying the LM volume fraction (*ϕ* = 10%, 20%, 30%) and FS weight fraction (*ψ* = 0%, 4%, 8%), and holding planetary mixing parameters constant, we isolate the role of rheology in shaping LM composite microstructure (Figure 1c,d). Subsequent microstructural analysis of the composites with microCT measurements enables 3D analysis to determine settling effects within LM composites by identifying individual droplets (Figure 1e,f) and isolating specific subsets of droplets by their diameter and position within the material, as demonstrated graphically in Figure 1g. This integrated approach enables not only visualization but also quantitative 3D characterization of thousands of droplets, establishing direct connections between processing conditions, rheological behavior, and resulting microstructure. Furthermore, through both experimental and modeling approaches, we can predict and design LM microstructure homogeneity to achieve a ≈ 10^5^x increase in electrical conductivity upon indentation. By promoting or suppressing LM droplet settling and agglomeration, we can tailor LM microstructures to improve composite performance in functional devices.

## 2 Results and Discussion

### 2.1 Materials and Processing

To investigate how rheology influences LM composite microstructure, we prepared materials by dispersing eutectic gallium–indium (EGaIn) into a PDMS matrix with FS as a rheological modifier. Planetary mixing produced emulsions that were cast and cured, with mold sizes selected to facilitate either optical imaging or high‐resolution microCT characterization. Varying the LM and FS content generated composites spanning a wide range of viscosities, which directly influenced droplet size, settling, and dispersion. For rheological measurements, samples were prepared without curing agent to isolate flow behavior, with supplemental data (Figure S1, Supporting Information) confirming negligible differences relative to uncured mixtures.

### 2.2 Effects of Rheological Modification

**FIGURE 2:**
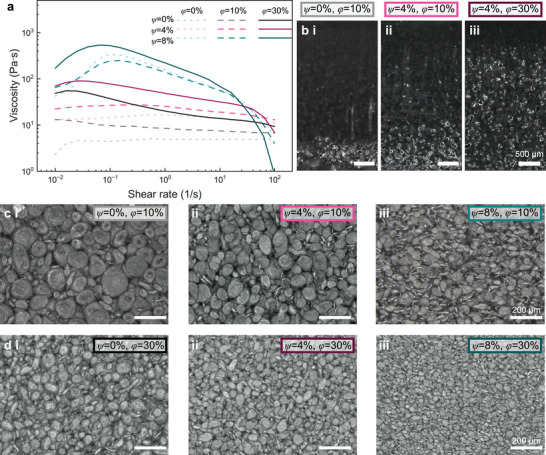
Rheological effects on LM microstructure. a) Shear rheology data summarizing the relationship between LM volume percent (*ϕ*), FS weight percent (*ψ*), and viscosity. b) Optical microscopy of LM dispersion due to settling in samples of i) *ψ* = 0%, *ϕ* = 10%, ii) *ψ* = 4%, *ϕ* = 10%, and iii) *ψ* = 4%, *ϕ* = 30%. c) Optical microscopy of LM droplet microstructures for samples of *ϕ* = 10% with i) *ψ* = 0%, ii) *ψ* = 4%, and iii) *ψ* = 8%. d) Optical microscopy of LM droplet microstructures for samples of *ϕ* = 30% with i) *ψ* = 0%, ii) *ψ* = 4%, and iii) *ψ* = 8%.

Changes in the concentration of LM (*ϕ*, vol.%) and FS (*ψ*, wt.%) in an uncured PDMS mixture will change the rheological properties. ^[^ ^41^ ^]^ The hydrophobic FS particles (≈ 16 nm) act as a rheological modifier by creating a physical particle‐polymer network via the adsorption of PDMS chains on the high‐surface‐area silica. ^[^ ^54^ ^]^ **Figure** **2**a shows the rheological curves for a sweep of nine different cases, where *ϕ* = 0%, 10%, 30% and *ψ* = 0%, 4%, 8%. The formation of this network results in increased viscosity across the board, particularly at low shear rates (≈ 0.01 s^−1^) ^[^ ^54^ ^]^, which is essential for preventing LM sedimentation. Additionally, increasing *ψ* enhances shear‐thinning behavior. This occurs because the particle network, which resists deformation at rest, breaks down under higher shear rates (> ≈ 1 s^−1^) as chains disentangle, causing the viscosity to drop. Generally, when more content (LM and/or FS) is added to the mixture, the viscosity increases. When LM is added to the matrix (e.g., *ϕ* = 0%), the viscosity increases across all shear rates by nearly an order of magnitude for shear rates below 10^−1^. Additionally, for each set of samples for a given content of LM (*ϕ* = 0%, 10%, 30%), the viscosity increases with increasing *ψ*. Furthermore, as *ψ* increases to *ψ* = 8%, the shear thinning behavior of the mixture also increases.

These changes in viscosity strongly influence the resulting LM composite microstructure. One clear effect is on droplet settling, as shown in optical cross‐sections of cured composites in Figure 2b. The most pronounced settling condition, *ψ* = 0%, *ϕ* = 10%, is shown in Figure 2bi. The lack of FS combined with the low LM content results in a low mixture viscosity, providing little resistance against droplet settling. When FS is added, as shown in Figure 2bii, there is still notable settling, but more droplets remain suspended in the matrix. This shows that when additional LM is added to the material (as in Figure 2biii), the more uniform distribution of droplets is largely attributed to the resulting increase in viscosity.

Viscosity also dictates droplet size, which decreases with increasing *ψ*, and therefore, increasing viscosity, as shown in Figure 2c,d. For instance, in Figure 2ci (*ψ* = 0%, *ϕ* = 10%), many droplets exceed 200 µm in diameter, whereas at *ψ* = 4% and 8% the average droplet size decreases to ≈100 µm and below 100 µm, respectively. A similar reduction is observed across *ϕ* = 30% samples (Figure 2d). Comparisons between Figure 2c,d further confirm that, for a given *ψ*, droplet size decreases as LM content increases. The influence of *ψ* is particularly evident in Figure 2 ciii, diii, where enhanced shear forces increase the frequency of small LM droplets, with some slightly elongated. While optical microscopy reveals these effects of viscosity on the composite surface, it does not capture the full 3D structure, highlighting the need for further analysis to fully understand the composite microstructure.

### 2.3 MicroCT Characterization

**FIGURE 3:**
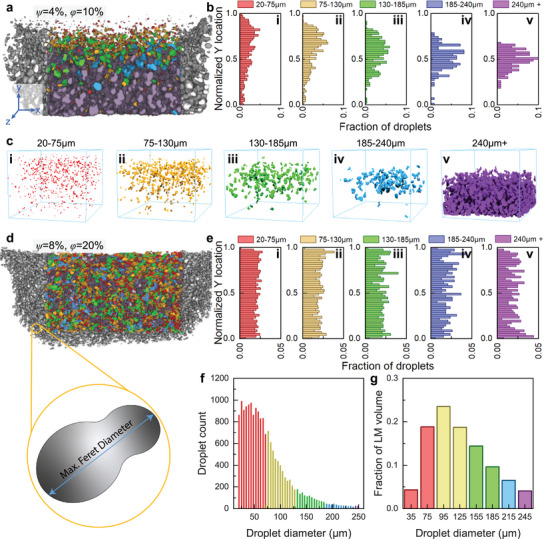
Representative microCT data collected for LM composites. a) MicroCT reconstruction of an LM composite with *ψ* = 4%, *ϕ* = 10%, colored to differentiate droplet/agglomeration size. b) Histogram representations of droplet y‐location with (i‐v) increasing diameter and c) Visualization of individual LM droplets in *ψ* = 4%, *ϕ* = 10% grouped by droplet diameter. d) MicroCT reconstruction of an LM composite with *ψ* = 8%, *ϕ* = 20%, colored to differentiate droplet/agglomeration size. e) Histogram representations of droplet y‐location with (i‐v) increasing diameter in *ψ* = 8%, *ϕ* = 20%. f) Histogram of maximum Feret diameter for and g) Volume‐weighted histogram for LM droplet populations in the *ψ* = 8%, *ϕ* = 20% composite samples.

For a more robust understanding of how processing impacts LM composite microstructure, we used microCT analysis. This technique enables 3D, non‐destructive microstructural evaluation with detailed statistics about individual LM droplets and the full droplet ensemble. Using thresholding and a Watershed transform analysis^[^ ^55^ ^]^, bright regions of the microCT scan are separated into individual droplets, enabling quantitative 3D analysis of its volume, diameter, and location in X/Y/Z space. Here, we focus on the droplet's maximum Feret diameter (farthest distance between two points of a droplet in 3D space), droplet volume, and settling behavior along the Y‐axis. Representative examples are shown in **Figure** **3**a (Video S1, Supporting Information) and Figure 3d (Video S2, Supporting Information) for samples of *ψ* = 4%, *ϕ* = 10% and *ψ* = 8%, *ϕ* = 20%, respectively, where droplet sizes are color‐coded, and an inset illustrates the definition of Feret diameter in 2D.

Settling behavior of droplets can be analyzed quantitatively through distribution plots (Figure 3b) and qualitatively with spatial renderings (Figure 3c) which both show the distribution of droplets along the Y‐axis (settling direction). The *ψ* = 4%, *ϕ* = 10% sample shows pronounced settling, with large purple droplets (indicating diameters > 240 µm) concentrated near the bottom of the sample. In fact, the majority of the LM volume is agglomerated at the bottom of the sample due to settling during curing. This behavior can be explained according to Stokes' law, where the velocity of a spherical particle in a fluid is proportional to its diameter squared.^[^ ^56^ ^]^ Thus, larger LM droplets will settle and agglomerate more quickly before the composite is fully cured. Consequently, droplets larger than 185 µm are rarely found in the upper quarter of the cured sample (Figure 3biii–v). Furthermore, when isolating sets of droplets of decreasing size, they are found to be more evenly dispersed throughout the sample. For example, smaller droplets (< 75 µm) remain well dispersed throughout the matrix (Figure 3bi,ii). MicroCT thus provides quantitative 3D visualization of LM droplet size and dispersion that is not possible with surface microscopy alone.

In contrast, the *ψ* = 8%, *ϕ* = 20% sample shown in Figure 3d has a uniform, homogeneous distribution of LM droplets. Unlike the sample with appreciable agglomeration observed in Figure 3a, this material has droplets of all sizes distributed throughout the sample, seen visually in Figure 3d (as well as Figure S2, Supporting Information) and graphically in Figure 3e. Even large droplets (185–240 µm, Figure S2f, Supporting Information) are suspended throughout, indicating that the increased viscosity of this formulation is sufficient to prevent settling, creating a homogeneous composite material.

To further assess droplet populations, we analyzed both unweighted and volume‐weighted distributions. The unweighted histograms (Figure 3f) display the relative counts of droplets by Feret diameter (20–250 µm), while weighted histograms (Figure 3g) account for droplet volume, highlighting subsets that dominate the overall LM content. In these plots, the droplet diameter measured is the maximum Feret diameter of the droplet, and the range analyzed is for diameters between 20 and 250 µm. Since a spherical droplet's diameter scales with volume as *d* ^3^ ∼ *V*, larger droplets contribute disproportionately to total LM content. Thus, we present the population distribution of droplets in both forms (i.e., weighted and unweighted histograms). From the data (Figure 3f,g), while many droplets fall below 70 µm in diameter, the volumetrically dominant subset lies between 80–100 µm, emphasizing the importance of weighted analysis for polydisperse droplet microstructures, which can provide more complete links between structure‐processing‐property relationships.

### 2.4 MicroCT Analysis

**FIGURE 4:**
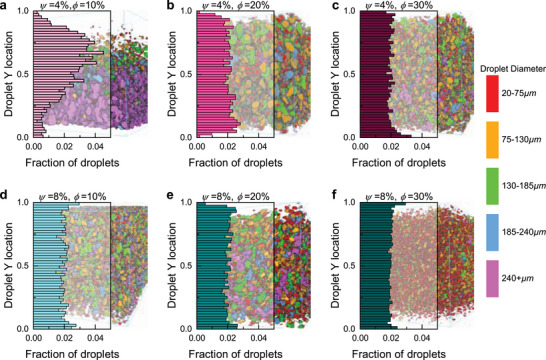
LM droplet settling in composites analyzed with microCT scanning. Vertical histograms of droplet dispersion overlaid on a microCT reconstruction for a) *ψ* = 4%, *ϕ* = 10%, b) *ψ* = 4%, *ϕ* = 20%, c) *ψ* = 4%, *ϕ* = 30%, d) *ψ* = 8%, *ϕ* = 10%, e) *ψ* = 8%, *ϕ* = 20%, and f) *ψ* = 8%, *ϕ* = 30%. In all microCT reconstructions, droplets are color‐coded by size: 20–75 µm (red), 75–130 µm (yellow), 130–185 µm (green), 185–240 µm (blue), and >240 µm (purple).

The LM droplet settling behavior, as introduced in Figure 2b and quantified in Figure 3a,b, is visualized in **Figure** **4** for samples ranging from *ϕ* = 10%, 20%, 30% and *ψ* = 4%, 8%. In Figure 4a, the histogram indicates a large number of droplets in the upper half of the volume and fewer in the lower half. However, the microCT rendering indicates substantial LM agglomeration near the bottom, with the top being more sparse. This discrepancy highlights how a small number of large, high‐volume droplets can dominate the lower region and create composite inhomogeneity.

In contrast, the other samples show more homogeneous vertical distributions. For example, Figure 4b,c suggests that *ψ* = 4% samples with *ϕ* = 20%, 30% exhibit only a slight increase in droplet count near the bottom 10% of the composites, with relative frequency values rising just above 0.02. Compared to Figure 4a these deviations are minor, and overall the histograms in Figure 4b–f confirm that *ϕ* = 20%, 30% with *ψ* = 4%, as well as all *ψ* = 8% samples, display well‐dispersed droplet populations. The high viscosity for *ψ* = 8% is sufficient to suspend droplets of all sizes during curing, resulting in uniformly homogeneous composite.

Recalling the rheological data from Figure 2a, the only sample with *ψ* > 0% that shows pronounced settling is *ψ* = 4%, *ϕ* = 10%. At a shear rate of 10^−1^ 1/s, this mixture has a viscosity of about 20 Pa·s, while the next‐most viscous case (*ψ* = 4%, *ϕ* = 30%) is nearly 100 Pa·s. The stark difference in microstructure between these two samples indicates that the critical viscosity for suppressing settling lies within this range. Beyond a viscosity of roughly 100 Pa·s, increased *ψ* results in incrementally more homogeneity, providing a metric for homogeneous LM dispersions for these compositions.

**FIGURE 5:**
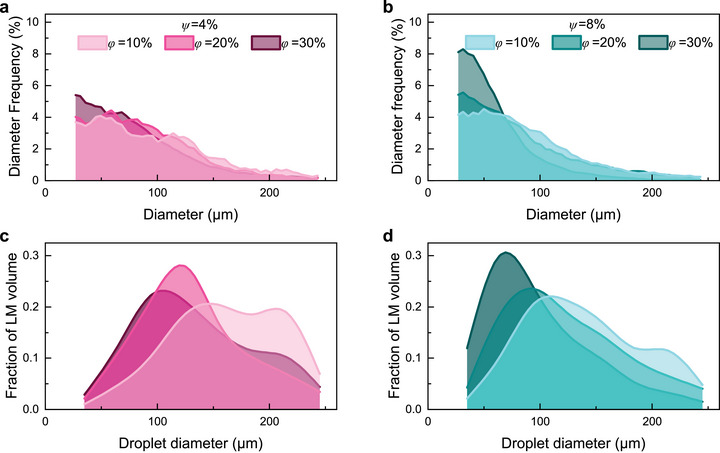
Statistical analysis of LM droplet sizes. a) Histogram data for LM composites with *ψ* = 4%, with darker shade indicating higher *ϕ*. b) Histogram data for LM composites with *ψ* = 8%, with darker shade indicating higher *ϕ*. c) Weighted histogram data for LM composites with *ψ* = 4, with darker shade indicating higher *ϕ*. d) Weighted histogram data for LM composites with *ψ* = 8%, with darker shade indicating higher *ϕ*. Trends in histogram bin data smoothed (a‐b with three‐bin moving average, c‐d with spline fit) with a line to guide the eye.

To characterize LM droplet size across all samples microCT scanned, histograms are presented in **Figure** **5** (total droplet counts and cumulative droplet volumes provided in Table S1, Supporting Information). Overall, we find that the influence of viscosity on droplet size distributions is consistent with existing work, where high shear forces lead to droplet breakup ^[^ ^57^ ^]^. Examining droplet count (Figure 5a,b), we observe that increasing viscosity produces narrower distributions centered on smaller diameters. The enhanced shear forces acting against droplet surface tension leads to greater droplet elongation and breakup during mixing, creating smaller, more uniform droplets ^[^ ^26^ ^]^. This effect is subtle at *ψ* = 4% (Figure 5a), where curves shift modestly from *ϕ* = 10% to *ϕ* = 30%, but pronounced at *ψ* = 8% (Figure 5b), where the broad distribution of *ϕ* = 10% becomes sharp and narrow at *ϕ* = 30%. Higher *ψ* also narrows distributions overall, with fewer droplets above 100 µm, consistent with enhanced shear forces breaking up larger agglomerates.

Weighted histogram data in Figure 5c,d indicate the volumetrically prevalent LM droplet sizes within composites, in contrast to population histograms. As *ϕ* increases, smaller droplets account for a greater volume within the composite, shifting the peak toward smaller droplet diameters. For both *ψ* = 4%, 8%, *ϕ* = 10% samples have distribution peaks furthest to the right, indicating that high‐volume droplets dominate the effective population of LM. At *ψ* = 4%, *ϕ* = 10% shows no clear peak, with nearly 60% of LM in 140–230 µm droplets–likely agglomerates formed by settling. In contrast, *ϕ* = 20% and 30% samples peak at 110–140 µm and 80–110 µm, respectively. A similar progression occurs at *ψ* = 8%, where peaks shift further left to 110–140 µm (*ϕ* = 10%), 80–110 µm (*ϕ* = 20%), and 50–80 µm (*ϕ* = 30%). Thus, increasing viscosity from *ψ* = 4% to 8% drives a greater volumetric prevalence of smaller droplets.

The peaks seen in Figure 5c,d are uniquely useful for understanding the droplet population in a sample. While the statistics seen in Figure 5a,b are helpful in showing the relative quantities of droplets in a sample, they fail to show which subset of droplets accounts for the greatest volume fraction. Many droplets may be smaller than 50 µm, but droplets near 100 µm can control the bulk LM content due to their volume. As the volume fractions of different droplet sizes change, the droplet packing fractions and coordination numbers may be impacted^[^ ^58^ ^]^, which may ultimately lead to a change in properties.^[^ ^59^ ^]^ Therefore, understanding how trends in droplet volume, relative to number frequency, impacts properties is essential for linking microstructure to properties.

### 2.5 Modeling LM Dispersion

To support the experimental results, we utilize a quantitative model to predict microstructure homogeneity, specifically the droplets stratification through the thickness, as a means to design desired microstructures. From the rheology experiments in Figure 2, it is clear that parameters such as *ψ* and *ϕ* strongly influence the viscosity of the uncured composite. Because the high‐speed mixing process generates a distribution of droplet sizes (Figure 5), these rheological properties directly shape the resulting microstructure, including how the droplets stratify and settle across the sample thickness. According to Stokes' Law, the settling velocity of a particle in a fluid scales with the square of its diameter.^[^ ^56^ ^]^ By quantifying *ψ* and *ϕ* and linking them to droplet size distributions, particle settling models can be used to predict microstructural homogeneity.

Our droplet‐settling model is adapted from Shojaei and Arefinia,^[^ ^60^ ^]^ which incorporates the time‐dependent deceleration of particle sedimentation during polymer curing, as shown in Equation (1).

(1) $$u=\left(1-\phi \right)\frac{d^{2}\left(\rho_{LM}-\rho_{p}\right)g}{18\mu_{p}\left(0\right)}\mu_{r}^{-1}\exp\left(-kt\right)$$

This equation modifies Stokes' Law, which defines the relationship for *u* as proportional to diameter squared (*d* ^2^), the difference in LM and polymer density (*ρ*_*LM*_, *ρ*_*p*_, respectively), and gravity constant (*g*), and inversely proportional to the initial viscosity of the polymer (μ_*p*_(0)). The first modification addresses inter‐droplet interactions by relating LM concentration to *u*, necessitating the $\left(1-\phi \right)\mu_{r}^{-1}$ correction factor (*ϕ*, *μ*_*r*_, being LM volume fraction and relative viscosity between the suspension and pure polymer, respectively). Furthermore, the exp (−*kt*) term accounts for the increasing polymer viscosity with cure time, where *t* is cure time *k* is a rate constant for viscosity increase.

**FIGURE 6:**
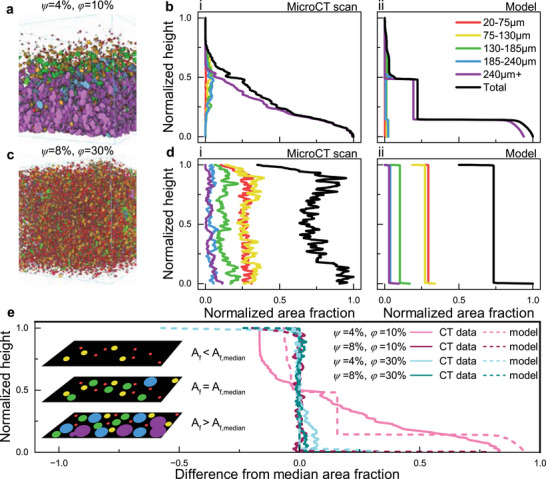
Modeling LM droplet settling. a) MicroCT scan of a *ψ* = 4%, *ϕ* = 10% sample, with LM droplets color‐coded by size. b) Normalized area fraction of droplets across the sample height for *ψ* = 4%, *ϕ* = 10%, as (i) measured from microCT scanning and (ii) computed by the model. c) MicroCT scan of a *ψ* = 8%, *ϕ* = 30% sample, with LM droplets color‐coded by size. d) Normalized area fraction of droplets across the sample height for *ψ* = 8%, *ϕ* = 30%, as (i) measured from microCT scanning and (ii) computed by the model. e) Measure of microstructure homogeneity across samples, from both experimental microCT and modeled data, comparing the difference from median area fraction. Inset visually illustrates what distance from median area fraction means at a given height, dY.

The model was evaluated in MATLAB. The initial viscosity of the matrix, *μ*_*p*_(0), was determined from the rheology data shown in Figure 2a, and the curing rate constant, *k* was fitted by linearizing existing PDMS curing kinetics data^[^ ^61^ ^]^. While the increased filler content was found to have a slight effect on curing rate, this did not have a notable effect on predicted microstructure (as shown in Figure S3, Supporting Information). Relative viscosity was determined locally using the empirical model proposed by Arefinia and Shojaei^[^ ^60^ ^]^, shown in Equation (2)

(2) $$\mu_{r}={\left(\frac{\phi_{m}}{\phi_{m}-\phi }\right)}^{a(\phi /\phi_{m})+b}$$

where *ϕ*_*m*_ is the maximum droplet packing fraction, and *a* and *b* are parameters defined as ‐0.3 and 2, respectively. The volume was discretized into 801 grid points, and boundary conditions were set such that mass is conserved in the system. The flux and velocity of the droplets were numerically determined like the scheme in Arefina and Shojaei. Our program, however, supports an arbitrary number of droplet classes, which was set to five in this model, so the population of droplets were binned into five representative diameters. The results of our model compared to experimental data are found in **Figure** **6**.

Figure 6 provides both visual and quantitative comparisons of *ψ* = 4%, *ϕ* = 10% (Figure 6a,b) and *ψ* = 8%, *ϕ* = 30% (Figure 6c,d). The area fraction plotted along the x‐axis corresponds to the 2D fraction of each slice occupied by droplets at a given height, Y, normalized to the range of area fractions measured for each sample. Examples of cross‐sectional slices where area fraction is obtained are shown in Figure S4 (Supporting Information). For the low‐viscosity sample (graphed in Figure 6b), the large agglomerates (240 µm+) dominate the microstructure, leading to a high degree of settling in experimental and modeled data. The model captures this stratification but exhibits more abrupt transitions between settled and unsettled regions, reflecting its discrete droplet size treatment rather than the continuous size distribution observed experimentally. In contrast, the high‐viscosity sample (graphed in Figure 6d) has a more homogeneous distribution of droplets throughout the sample height, showing strong agreement with the model prediction.

To quantify microstructural homogeneity across all samples, we introduce a normalized area fraction parameter, Δ*A*_*f*_, to account for the deviation from median area fraction:

(3) $$\Delta A_{f}=\frac{A_{f}-A_{f,median}}{A_{f,max}-A_{f,min}}$$

where *A*_*f*_ represents the area fraction, *A*_*f*, *median*_ is the median for a given sample, and *A*_*f*, *max*_, *A*_*f*, *min*_ are the maximum and minimum *A*_*f*_ for all samples, respectively. The inset of Figure 6e illustrates how different *A*_*f*_ values relate to *A*_*f*, *median*_. The results demonstrate that samples with insufficient viscosity (e.g., *ψ* = 4%, *ϕ* = 10%) exhibit notable Δ*A*_*f*_ variations, indicating higher microstructural heterogeneity. Conversely, samples with adequate viscosity (e.g., *ψ* = 8%, *ϕ* = 10%) maintain Δ*A*_*f*_ values close to zero throughout the height, showing homogeneous droplet distribution. These results are supported by both experimental and modeling data, which provides a design tool for tailoring composite properties and a clear quantitative metric for microstructure heterogeneity.

### 2.6 Droplet Dispersion and Embossing

**FIGURE 7:**
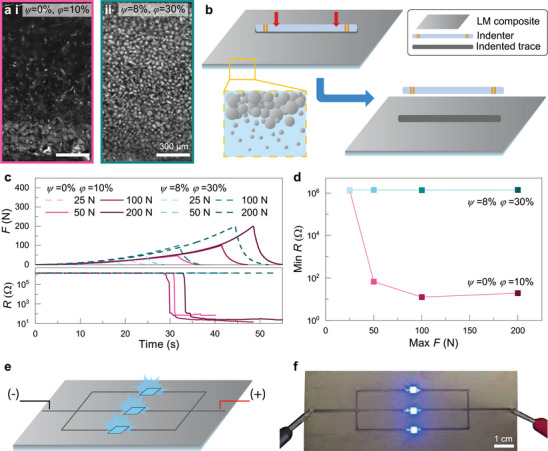
Droplet dispersion effect on electromechanical properties. a) Optical micrographs contrasting the different droplet dispersions seen in (i) *ψ* = 0%, *ϕ* = 10% and (ii) *ψ* = 8%, *ϕ* = 30%. b) Schematic of indentation test for in situ measurement of electrical resistance, with inset highlighting the settled microstructure of the *ψ* = 0%, *ϕ* = 10% sample. c) Electromechanical data for indentation LM composites, showing load applied and electrical resistance. d) Minimum resistance achieved for samples of *ψ* = 0%, *ϕ* = 10% and *ψ* = 8%, *ϕ* = 30%, with maximum load ranging from 25 to 200 N. e) Schematic of demonstration of electrical conductivity achieved with embossing in a settled LM composite. f) Image of functional LM composite circuit, fabricated by embossing a settled *ψ* = 0%, *ϕ* = 10% sample.

Changing the rheology of LM composite mixtures during processing has a significant impact on the microstructure, which ultimately affects the electromechanical properties of the material. To demonstrate this, we embossed two materials with different droplet dispersions to show how the electrical response to mechanical stress changes based on the microstructure. Specifically, we use *ψ* = 0%, *ϕ* = 10% and *ψ* = 8%, *ϕ* = 30% samples, which have drastically different droplet dispersions (**Figure** **7**a), to show how readily each composite activates, or how the droplets change from discrete electrically insulating droplets to electrically conductive percolated networks, under mechanical loading.

While indenting each sample with a cylindrical probe, as shown in the schematic of Figure 7b, the in situ measurement of electrical resistance was conducted. In both the well‐dispersed and highly settled cases, an indentation load of 25 N was insufficient to activate the material, as evident in Figure 7c where these cases did not see a drop in resistance. However, in each subsequent case (50–200 N) for the *ψ* = 0%, *ϕ* = 10% sample, the indentation trace became electrically conductive, as noted by a substantial drop in resistance. This did not occur for the *ψ* = 8%, *ϕ* = 30% sample at the same loads, which is shown by a constant, high resistance regardless of load. A summary of minimum resistance achieved from indentation experiments on each sample is shown in Figure 7d, demonstrating that a highly settled microstructure of LM droplets is more readily activated than a well‐dispersed sample. Because the microstructure exhibiting significant settling has a large volume of LM locally, the LM is more likely to percolate into an interconnected, electrically conductive network, as shown in optical microscopy in Figure S5 (Supporting Information). This formation of a percolated network allows for a huge drop in electrical resistance, about five orders of magnitude during embossing, enabling the formation of soft circuits. Alternatively, the well‐dispersed sample has a consistent volume fraction of LM homogeneously distributed throughout the sample, and despite having more liquid metal within the entire composite, it is not sufficiently densely packed to become percolated, maintaining its high electrical resistance. These results demonstrate how LM microstructure can dramatically impact electrical conductivity during composite processing. This provides key insight into how to tune material composition and fabrication to enable enhanced electrical conductivity of soft, LM composites for soft electronics and other deformable devices.

Embossing to form electrically conductive LM networks is useful for the on‐demand formation of LM circuits. For the, highly settled *ψ* = 0%, *ϕ* = 10% sample, embossing forms conductive LM circuits, which can illuminate three LED lights in parallel. However, the more uniformly dispersed *ψ* = 8%, *ϕ* = 30% sample does not become conductive after embossing. While this limits its use for conductive applications, such a microstructure could instead serve as a dielectric. The low volume loading of the *ψ* = 0%, *ϕ* = 10% sample is also favorable, providing guidance for how to process LM composites for electrical conductivity at much lower LM loadings than conventional well dispersed composites. These results highlight how processing‐driven changes in microstructure directly govern the electromechanical properties of LM composites.

## 3 Conclusion

This work provides a detailed, quantitative analysis of how processing conditions, specifically varying LM volume fraction and FS weight loading, govern droplet size distribution and dispersion in LM composites. Through microCT, we quantify thousands of microscale LM droplets in a composite that can be analyzed in 3D, revealing how viscosity governs droplet population statistics and settling behavior. Increasing the weight fraction of FS increases the viscosity of uncured LM composite, leading to greater homogeneity, smaller average droplet size, and reduced droplet polydispersity. By isolating subsets of droplet sizes, we capture degrees of LM droplet homogeneity throughout the composite that cannot be resolved with surface techniques like optical microscopy. These results are further supported by predictive modeling through a modified Stokes Law formulation, which provides a design tool for microstructural control. These insights establish direct links between rheology and microstructure, enabling formulations tailored for specific functions such as electrical activation. More broadly, this approach lays a foundation for systematically evaluating and optimizing LM composite properties through processing–structure relationships.

## 4 Experimental Section

### Materials

To fabricate composite materials, three components were mixed together. The LM material used was a eutectic gallium‐indium (EGaIn), which was alloyed as 75% Ga and 25% by weight. The matrix material, polydimthylsiloxane (PDMS, Dow Sylgard 184), was mixed in a ratio of 10:1 base to curing agent. The rheological modifier, fumed silica (FS, lateral size ≈ 16 nm, hydrophobic, Eastchem), was added as an inert material to tune viscosity and increase the shear‐thinning behavior of the LM composite mixture. The content of LM and FS were varied to obtain different material viscosities and, ultimately, different material microstructures.

### Processing

Base and curing agent were added to an SC 60 mixing cup in a 10:1 ratio and mixed in a planetary centrifugal mixer (DAC 1200–500 VAC, FlackTek speed mixer) under vacuum. To modify the rheology, FS was measured into the mixing cup and suspended in the mixture using the centrifugal mixer. Then, EGaIn was incorporated into the PDMS/FS by first suspending the LM by hand, then mixed in the centrifugal mixer at 1000 RPM for 1 min. After fully combining the components of the composite, the material was cast into an acrylic mold and cured in a convection oven at 80 °C overnight.

Two sizes of mold were used in the processing of these materials. Wider molds (40x60 mm^2^) were used for optical microscopy, imaging both the top and bottom surfaces as well as for cross‐sectional imaging. Small molds (1x2 mm^2^) were used for producing samples for microCT scanning to limit the post‐casting manipulation of samples. All samples were cast into acrylic with a thickness of 6.4 mm.

For samples processed for rheological testing, curing agent was omitted to ensure that rheological data was not influenced over time by curing. As shown in Figure S1 (Supporting Information), there was a slight reduction in the viscosity of mixture without curing agent and with curing agent added immediately before. However, after three hours of curing at ambient conditions, the composite material with curing agent shows non‐negligible increases in viscosity as compared to material without curing agent.

### Rheological Analysis

The rheological properties of both pristine PDMS and PDMS loaded with LM and FS were analyzed using an HR‐30 rotational rheometer (TA Instruments). Measurements were performed with stainless steel parallel plates (25 mm in diameter) set at a fixed 1 mm gap. Viscosity was assessed via a flow sweep test across shear rates ranging from 0.01 to 100 s^−1^. All tests were conducted at ambient conditions (approximately 25°C).

### MicroCT Scanning and Analysis

MicroCT scanning was performed on a Skyscan 1172 benchtop microCT scanner. The pixel size used for imaging was 4 µ*m*, and the beam settings were set to 88 kV of voltage and 114 µ*A* of current at 10 W power.

Dragonfly software was used to visualize and analyze microCT scans. Histogram cropping was used to reduce noise and Watershed Transform analysis was used to identify individual LM droplets.

## Conflict of Interest

The authors declare no conflict of interest.

## Supporting information

Supporting Information

Supporting Information

Supporting Information

## Data Availability

The data that support the findings of this study are available from the corresponding author upon reasonable request.
