# Supplementary material for: Linking Viscosity and Droplet Microstructure in Liquid Metal Composites via 3D MicroCT Analysis
Source: Small. 2026 Jan 12;22(9):e12413. doi: 10.1002/smll.202512413 (PMC12895225; doi:10.1002/smll.202512413)
Supplement: Supplementary file 1 — Supporting Information [file SMLL-22-e12413-s003.pdf]

NANO · MICRO small

Supporting Information

for *Small*, DOI 10.1002/smll.202512413

Linking Viscosity and Droplet Microstructure in Liquid Metal Composites via 3D MicroCT Analysis

*Hugh P. Grennan, Ohnyoung Hur and Michael D. Bartlett**

Supplementary Information

Linking Viscosity and Droplet Microstructure in Liquid Metal Composites via 3D MicroCT Analysis

Hugh P. Grennan[1], Ohnyoung Hur[1], and Michael D. Bartlett[1,2*]

[1]Mechanical Engineering, Soft Materials and Structures Lab, Virginia Tech, Blacksburg, VA 24061, USA.

[2]Macromolecules Innovation Institute, Virginia Tech, Blacksburg, VA 24061, USA.

[*]Corresponding author email: mbartlett@vt.edu

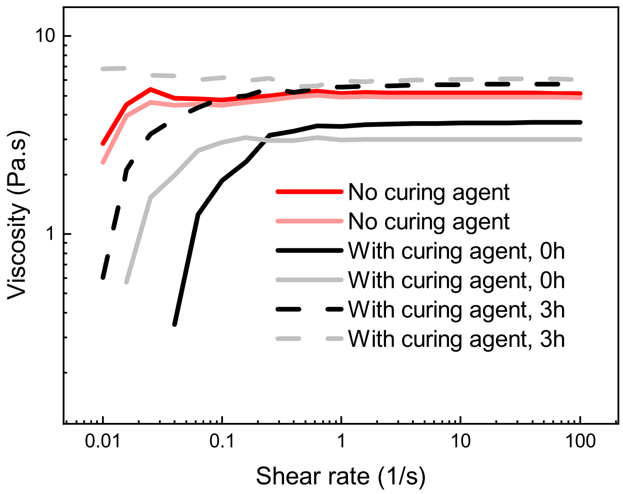

Fig. S1 Rheological data for unfilled PDMS with and without curing agent.

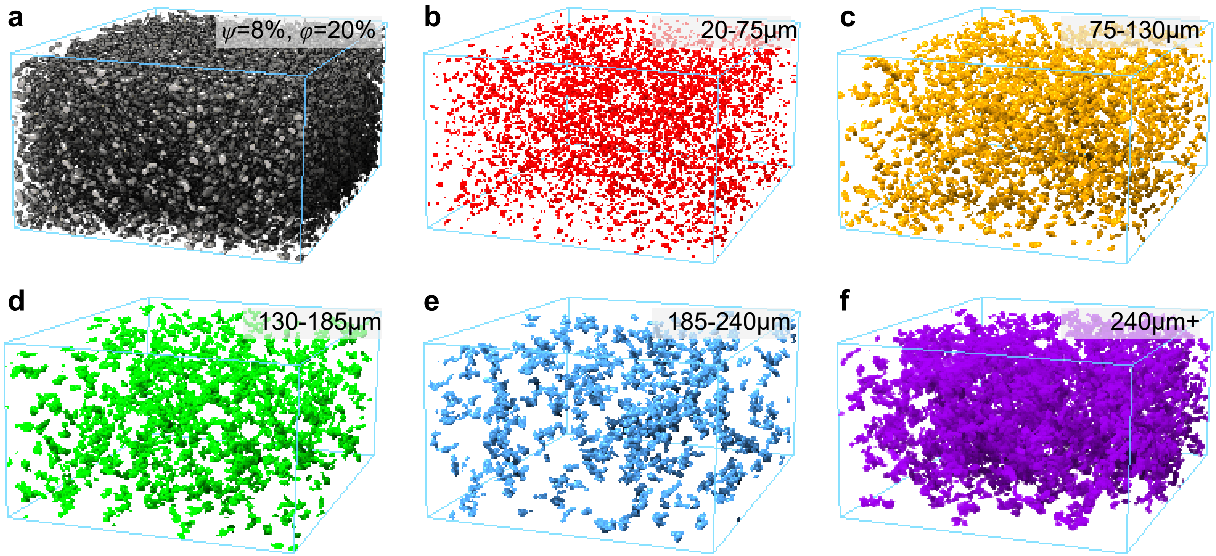

Fig. S2 MicroCT analysis of an LM composite of $\psi = 8\%$, $\phi = 20\%$. **a)** Raw microCT rendering of LM composite. **b-f)** Spatial arrangement of droplets with diameters of **b)** 20-75 μm, **c)** 75-130 μm, **d)** 130-185 μm, **e)** 185-240 μm, and **f)** 240 μm+.

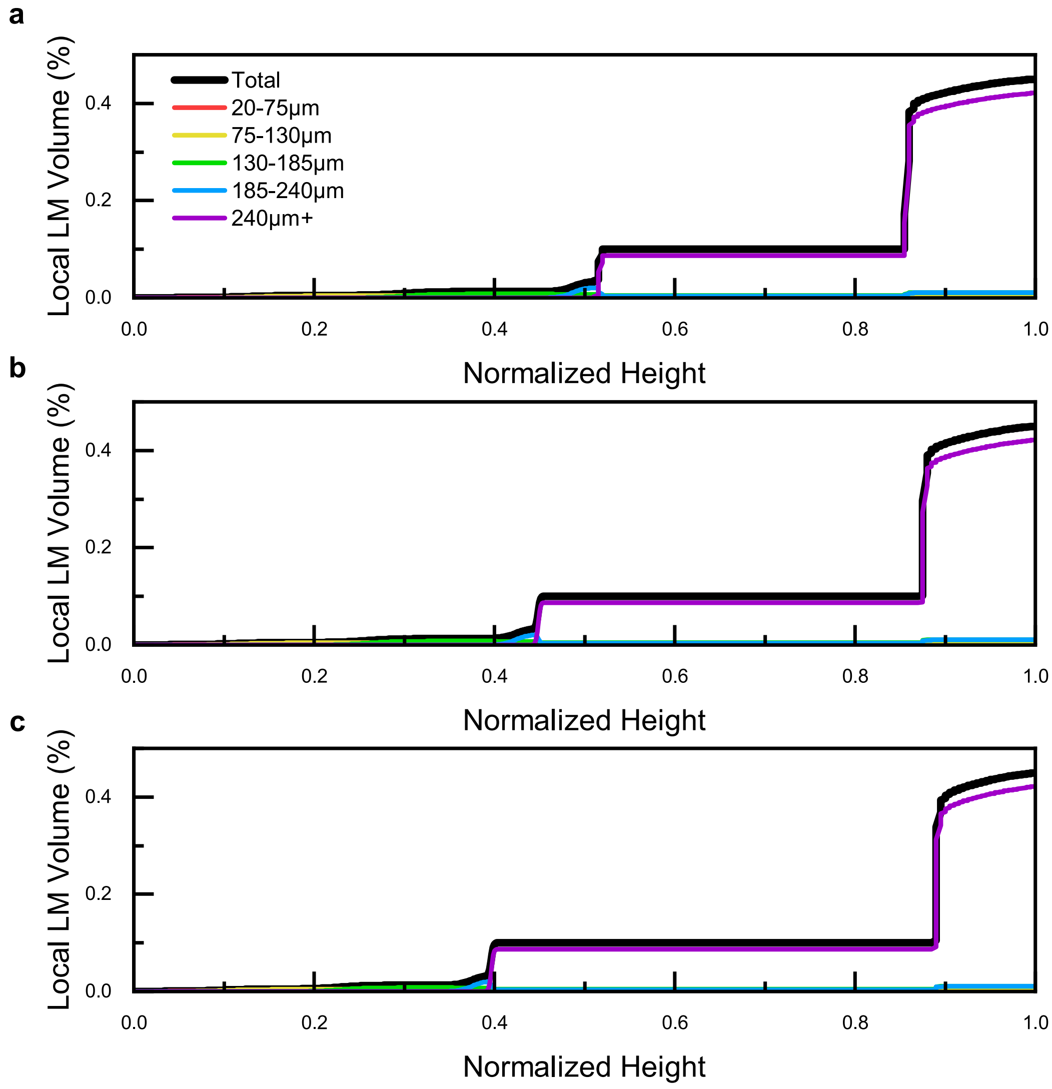

Fig. S3 Changes in curing rate constant, k, by up to 30% have minimal effects on microstructure prediction. The predicted distribution of droplets in a sample of $\psi = 4\%, \phi = 10\%$ with a k of **a)** 0.3 (original), **b)** 0.345 (15% increase), and **c)** 0.390 (30% increase).

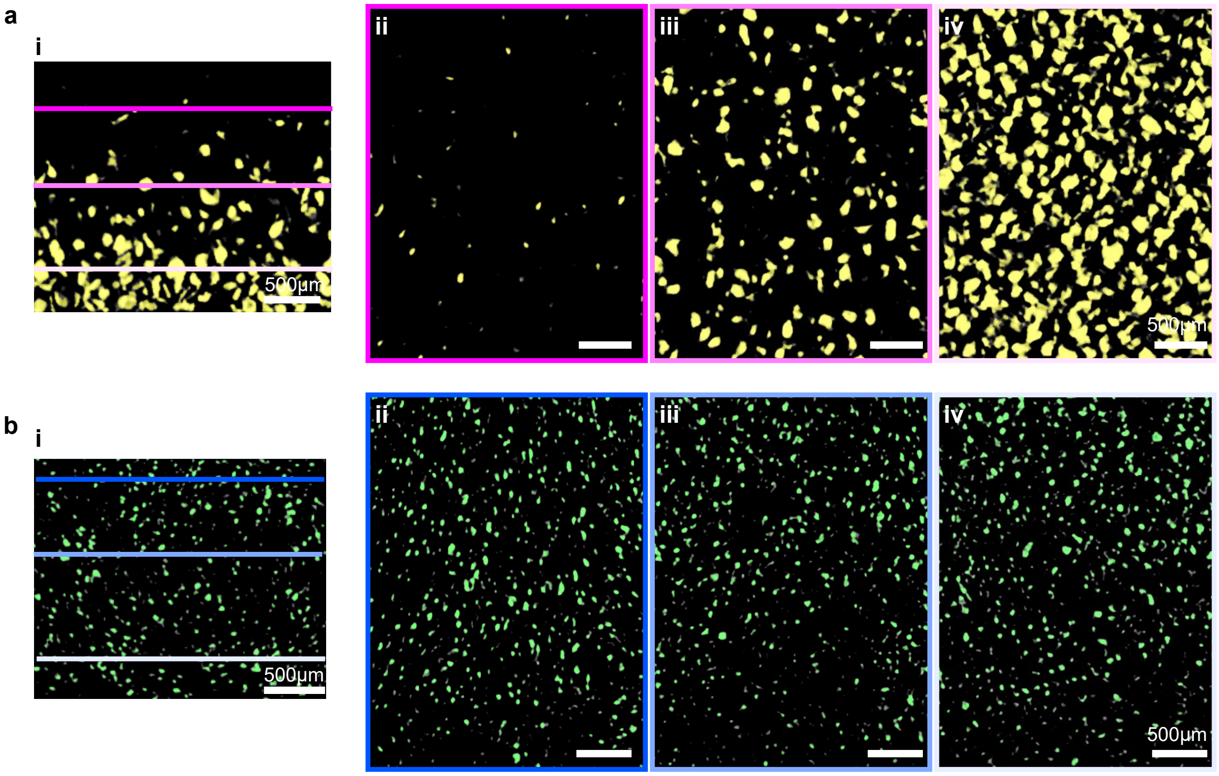

Fig. S4 Determining area fraction from MicroCT scans. MicroCT cross sectional slices of **a)** $\psi = 4\%$, $\phi = 10\%$ and **b)** $\psi = 8\%$, $\phi = 30\%$. Three Y-values, noted in i), are shown in ii-iv) to show degree of homogeneity.

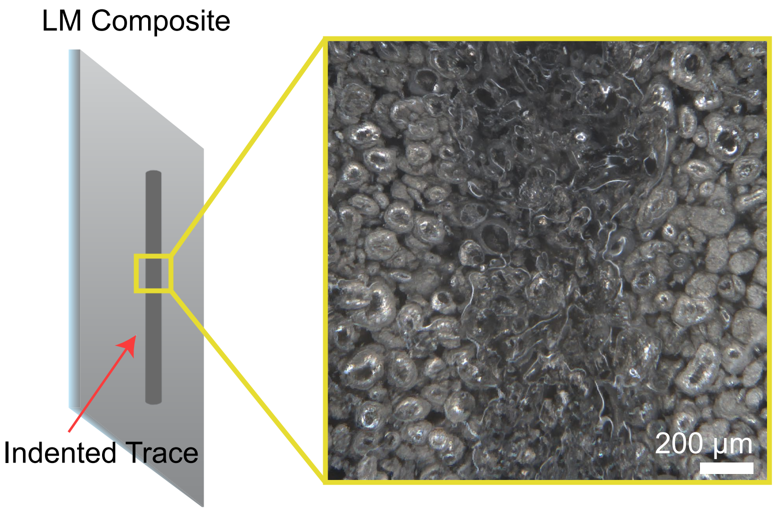

Fig. S5 Optical microscopy of electrically conductive indented LM trace.

Table S1: Droplet count, cumulative droplet volume, and total volume over which droplets were measured for each composite.

Filler Composition (ϕ/ψ)	Number of Droplets	Cumulative Droplet Volume (mm^3)	Total Measured Volume (mm^3)
10/4	2137	2.67	20.11
20/4	4265	0.40	7.37
30/4	11062	1.58	16.01
10/8	6742	0.73	12.82
20/8	9958	1.40	13.56
30/8	28205	0.79	17.58
